# Drosophotoxicology: An Emerging Research Area for Assessing Nanoparticles Interaction with Living Organisms

**DOI:** 10.3390/ijms17020036

**Published:** 2016-02-14

**Authors:** Mariana Carmen Chifiriuc, Attila Cristian Ratiu, Marcela Popa, Alexandru Al. Ecovoiu

**Affiliations:** 1 Microbiology Immunology Department, Faculty of Biology, University of Bucharest, 1–3 Portocalelor, Sector 5, Bucharest 060101, Romania; carmen_balotescu@yahoo.com (M.C.C.); bmarcelica@yahoo.com (M.P.); 2 Department of Genetics, Faculty of Biology, University of Bucharest, 1–3 Portocalelor, Sector 5, Bucharest 060101, Romania; attila.ratiu@bio.unibuc.ro

**Keywords:** *Drosophila melanogaster*, larvae, adults, nanoparticles, drug-delivery, toxicity, mutant, behavior

## Abstract

The rapid development of nanotechnology allowed the fabrication of a wide range of different nanomaterials, raising many questions about their safety and potential risks for the human health and environment. Most of the current nanotoxicology research is not standardized, hampering any comparison or reproducibility of the obtained results. Drosophotoxicology encompasses the plethora of methodological approaches addressing the use of *Drosophila melanogaster* as a choice organism in toxicology studies. *Drosophila melanogaster* model offers several important advantages, such as a relatively simple genome structure, short lifespan, low maintenance cost, readiness of experimental manipulation comparative to vertebrate models from both ethical and technical points of view, relevant gene homology with higher organisms, and ease of obtaining mutant phenotypes. The molecular pathways, as well as multiple behavioral and developmental parameters, can be evaluated using this model in lower, medium or high throughput type assays, allowing a systematic classification of the toxicity levels of different nanomaterials. The purpose of this paper is to review the current research on the applications of *Drosophila melanogaster* model for the *in vivo* assessment of nanoparticles toxicity and to reveal the huge potential of this model system to provide results that could enable a proper selection of different nanostructures for a certain biomedical application.

## 1. Introduction

The fast emergence of nanotechnology allowed the obtaining of a wide range of different nanoparticles (NPs) for specific applications in the pharmaceutical, cosmetic, and other biomedical product industries, as wells as for developing imaging diagnosis techniques and photothermal therapy. However, many questions arise regarding safety and latent risks for human health and environment [1,2]. Taking into account that the huge potential of NPs for different applications is the result of the fact that they are more reactive than conventional-sized particles, it is also possible that they may also exhibit a higher cytotoxicity.

Most of the current nanotoxicology research uses *in vitro* models that do not offer information about the fate of NPs in the host organisms (biodistribution, accumulation, metabolism, persistence, elimination *etc.*) [3]. The *in vivo* studies are using mostly aquatic organisms, such as rainbow trout (*Oncorhynchus mykiss*), zebra fish (*Danio rerio*), nematode (*Caenorhabditis elegans*), algae, and daphnids [4,5,6,7]. Additionally, the protocols used in different studies for assessing the nanotoxicity are not standardized regarding the many variables occurring in this field of research (variations in size, fabrication procedures, aggregation, solubility, intracellular uptake, and cellular and animal models). These aspects impede on any comparison or reproducibility of the obtained results, raising the necessity of standardization and of setting up *in vitro*/*in vivo* experimental models for the characterization of NPs cytotoxicity and biocompatibility. Establishing of various standard pharmacological parameters, such as dosage, administration route, metabolism, *etc.* [8,9,10] is also required. 

## 2. Advantages of the *D. melanogaster* Experimental Model

*D. melanogaster* represents a genetically tractable model for many fields of biology. It offers an abundance of advantages, such as fast offspring turnover, affordable maintenance cost comparative to other invertebrate and vertebrate models, advanced genetic analyses tools, considerable gene homology with other organisms, including humans, and numerous options for detecting induced or abnormal phenotypes. For all these reasons, *D. melanogaster* is the model of choice for mutagenesis screens and for assessing the biological activity of different chemical compounds, including NPs [11,12]. Comparative to mammalian genomes, the *D. melanogaster*’s genome has a simpler structure, being prone to complex reverse genetics due to the availability of whole-genome RNAi libraries, molecular markers and cutting-edge genetic analysis tools. In contrast with *C. elegans*, which is a very simple experimental model containing only 959 somatic cells, *D. melanogaster* has fewer genes, but twice as many human homologs [13] and over 75% of human disease genes have homologs in *D. melanogaster*, some sharing more than 90 percent nucleotide sequence identity [14]. Major aspects regarding the use of *D. melanogaster* as a human diseases model, as well as comparisons with other animal models, such as *D. rerio* and *C. elegans*, are reviewed elsewhere [15].

The increasing interest for using *D. melanogaster* in toxicity studies motivated the proposal of a research field called drosophotoxicology [16,17]. The toxicity can be assessed in embryonic, larval, pupal, and adult developmental stages of *D. melanogaster* [18], and its response to NPs has been shown to be similar to that observed in mammalian models. Moreover, as the generation time of *D. melanogaster* under standard culture conditions at 25 °C is about 10 to 12 days from egg to adult, this nanotoxicology model allows to directly assess the impact of nanoparticle toxicity on the behaviour or development [16].

In drosophotoxicology, NPs are generally delivered by ingestion, but also by injection and inhalation, the last one having a great potential as a model procedure to study the effects of nanoparticle toxicity to the respiratory system [16,19]. The fly airway system shows many similarities to mammals, regarding the physiology and reaction towards pathogens, and is used as an animal model in asthma-related diseases. Its external tracheal openings are large enough to allow NPs entrance [20]. Exposure of the embryonic tissues can be achieved through *in vitro* incubation, injection, or by maternal feeding [17]. 

The motor and sensory behaviors of the larvae (foraging, chemo- and phototaxis) and of adults (flight, chemo-, photo- and geotaxis, courtship and mating, aggression, and grooming) can be evaluated using *D. melanogaster* model. In mutagenesis screens, the lethality rate, number of disrupted embryo, adult wing, or eye morphology (frequently evaluated by means of somatic mutation and recombination test (SMART) [21]), the incidence of widely known mutant phenotypes (e.g., hedgehog, wingless and Notch), and the reporter gene assay can be evaluated. Therefore, *D. melanogaster* model could bring an essential contribution to the nanotoxicology field and is expected to complement, or even replace, other animal models used to detect the NPs short-term and long-term toxicity and to elucidate the respective molecular mechanisms. The molecular pathways impacted by NPs could be elucidated using genetically sensitized fly strains for signaling pathways similar to vertebrates, such as Wnt [22], TGFβ [23], hedgehog [24], EGF [25], cytokine [26], and Notch [27] pathways. *D. melanogaster* could be efficiently used for throughput assays data generation due to several advantages offered by different scoring systems of specific phenotypes and behaviors. For example, recording of visible markers, such as green fluorescent protein (GFP), and obvious phenotypes (live/dead) are suitable for high throughput approaches, while monitoring of locomotor and circadian activity, learning, memory, aggression, and courtship are appropriate for medium or low throughput analyses [16,28,29].

Some authors have proposed a systematic and reproducible evaluation of NP toxicology in living systems by using the *D.* *melanogaster* model [30]. This approach would rely on the physical assessment and quantification of some key parameters that indicate the toxic effects of NPs, in an endeavor aiming to determine a certain value for the “toxicity factor”. The systematic classification of the toxicity levels of different nanomaterials is expected to lead to important developments in risk assessment and regulatory approval, as well as in extending the range of nanomedicine applications [31]. For this kind of application, the injection or direct microtransfer approaches are preferred to ingestion protocols because of the small and variable amounts of the ingested food as well as to the lower stability of the nanomaterials contained in food. The direct microtransfer is a uniform and more accurate assessment that allows the use of known concentrations in the nanogram range and permits a high number of replicates, as well as the identification of the specific stage in which mortality occurs. This methodology could also allow to establish quantitative parameters of the toxic effect, such as minimal toxic dose, acuteness of toxic effect, maximum allowable concentration in the environment, and the influence of surface modification on the toxicity. Such parameters can be very useful in tailoring the nanomaterial physicochemical properties in order to minimize their side effects. If fluorescently labeled, the nanomaterial can be traced across the life cycle in the surviving embryos. The use of transgenic flies with fluorescent markers for caspase 3 (indicating apoptosis), lactate dehydrogenase (indicator of necrosis), intact lysosomes, and reactive oxygen species (stress response) will provide additional mortality markers and will allow the localization of a certain toxic effect into a specific organ and/or system. *D. melanogaster* model could also be used to simultaneously assess the effectiveness of different nanomaterials in the treatment of human diseases or for understanding their molecular mechanisms [32].

## 3. Investigation of NPs Toxicity and Genotoxicity Using *D. melanogaster* Model

Among the first NPs tested for their cytotoxicity on *D. melanogaster* model were carbon nanotubes [33,34]. Dietary uptake of fullerene C_60_, carbon black, or single-walled or multi-walled nanotubes, fed to the larval stage, had no deleterious effect on egg-to-adult survivorship, although these nanomaterials are incorporated in tissues. When administered to adults, carbon black or single-walled nanotubes proved an intensive capacity of adherence to fly body surface, impairing the grooming behavior and locomotion and inducing increased mortality. These results show that nanomaterial superstructure and aggregation state influence its toxicity, and the adhesion of NPs to the fly body surface activates the grooming behavior leading to the nanoparticle transport inside the body [35].

Gallium phosphide (GaP) nanowires ingested by *D. melanogaster* larvae and/or adults did not (i) accumulate in the fly tissues; (ii) stimulated the immune response; (iii) modify the gene expression; or (iv) affect the life span or the somatic mutation rate [36].

The effects of magnetite or iron oxide (Fe_3_O_4_) NPs capped/modified/coated with pristine citric acid and 3-aminopropyltriethoxylsilane in concentrations of 300–600 μg/g have been investigated using the *D. melanogaster* model. The uptake of Fe_3_O_4_ NPs caused a significant decrease in the female fecundity, and a developmental delay at the egg-pupae and pupae-adult transitions. Additionally, adult uptake of Fe_3_O_4_ NPs disturbed the oogenesis period, induced ovarian defects, delays in egg chamber development, reduced the eggs length and of the nurse cells. Furthermore, Fe, Ca, and Cu trace element imbalances, along the anterior-posterior axis of the fertilized eggs were found [37]. The titanium dioxide (TiO_2_) and silver (Ag) NPs have been shown to induce a decrease of survival rate and fecundity, delays in development and the occurrence of distinct phenotypes [38,39,40,41,42].

Using *D. melanogaster* model it has been shown that indium III coating (which modulates the quantum dots (QDs) bioaccumulation in the organism), also decreases the toxicity of QDs, as compared to that of cadmium selenide (CdSe)/zinc sulfide (ZnS) QDs, which could be related to the release of poisonous Cd^2+^ ions after *in vivo* degradation of QDs [43]. These coated QDs significantly affect the lifespan of treated *D. melanogaster* populations and induce a significant increase in reactive oxygen species level. Furthermore, these QDs induce severe genotoxic effects and an increased rate of apoptosis in *D. melanogaster* haemocytes [44].

The exposure of *D. melanogaster* larvae to different concentrations, relevant to oral exposure to humans (0.002–2 mg/L), of food grade E171 TiO_2_ has shown that the tested NPs do not affect survival and fecundity, but significantly increased the pupation time. Additionally, down-regulation of catalase and superoxide dismutase 2 genes expression in larvae, without transferring this defect to adults during metamorphosis was detected [45]. In another study, the *in vivo* genotoxic activity of different concentrations of TiO_2_ NPs (0.08 to 1.60 mg/mL) was investigated after ingestion by using the wing spot test and the comet assay in *D. melanogaster* third instar larvae. Although the TiO_2_ NPs accumulated and induced cytotoxic effects at the level of larval midgut and imaginal disc, they failed to induce genotoxicity. A significant increase in DNA damage, with a direct dose-response pattern, was observed for TiO_2_ NPs in larvae and adults, revealing the necessity of using more than one genetic end-point in the evaluation of the genotoxic potential of nanomaterials [46].

Different concentrations of Ag NPs were tested to assess their acute (10–100 mg/L) and chronic toxicity (5 mg/L). The acute toxicity was observed at 20 mg/L, at which 50% of the tested flies were unable to emerge from pupae and to finish the developmental cycle. The long-term exposure to Ag influenced the fertility of *D. melanogaster* only during the first three generations, followed by the occurrence of flies’ adaptability to the Ag NPs low concentration exposure [47]. Long-term exposure at low concentrations (0.1 and 1 μg/mL) of AgNPs, but not Ag ions, significantly shortened the flies’ lifespan [48]. The expression of GAL4 is up-regulated in response to ingestion of low AgNPs concentrations and the respective mutant line showed a significantly increased tolerance to AgNPs, but also to dry starvation probably due to the up-regulation of c-Jun N-terminal kinases (JNKs) signaling [49]. The effects of AgNPs at nonlethal concentrations up to 50 mg/L on wild type *D. melanogaster* fed with AgNPs supplemented food revealed that AgNPs did not influence the survival rate. Instead, the flies revealed a lighter body color due to the loss of melanin pigments in the cuticle, while fertility and vertical movement ability were compromised. These biological effects could not be attributed to the presence of Ag ions. The activity of copper (Cu) dependent enzymes, *i.e.*, tyrosinase (essential for melanin synthesis) and copper-zinc (Cu-Zn) superoxide dismutase was decreased, despite the normal level of Cu. These data could be a consequence of the fact that by being isoelectric with Cu ion, Ag ion is able to replace Cu therefore decreasing Cu-dependent activity [50]. The AgNPs of 4–42 nm, in concentrations of 250–1000 μg/mL did not modify the spontaneous frequencies of wing spots indicating the lack of mutagenic and recombinogenic activity. However, the tested AgNPs induced pigmentation defects and reduction in locomotor ability in adult flies [51]. Polysaccharide coated 10 nm Ag NPs were evaluated for their toxic effects on heat shock stress, oxidative stress, DNA damage and apoptosis in *D. melanogaster*, by using third instar larvae fed with a standard diet supplemented with AgNPs for 24 and 48 h. AgNPs up-regulated the expression of heat shock protein 70 and induced oxidative stress in *D. melanogaster*, as revealed by the increased levels of malondialdehyde, superoxide dismutase and catalase [52]. AgNPs also up-regulate the cell cycle checkpoint p53 and cell signaling protein p38 (both involved in the DNA damage repair) and increase the activities of caspase-3 and caspase-9, indicating a pro-apoptotic effect [32,52].

Gold (Au) NPs have been also proved to induce genotoxic effects in *D. melanogaster* [53]. *D. melanogaster* larvae fed with different concentrations of AuNPs proved to absorb them at the level of the fat body, increasing the lipid anabolism by stimulating the PI3K/Akt/mTOR signalling pathway and fatty acids synthesis [54]. By stimulating this pathway, AuNPs may promote nutrient uptake at cellular levels, as demonstrated by the higher sugar levels recorded in flies fed with AuNPs. The main activation site of PI3K/Akt seems to be the cellular membrane. The influence of AuNPs on the metabolic pathways opens the avenue for potential new applications of AuNPs, either alone or conjugated with different drugs in the management of metabolic disorders. Among cytotoxicity effects, a possible mutagenic effect was associated with AuNPs. The aberrant eye phenotype was called “nanomaterial-mutated” and was transmitted to the descendants [55]. Rare individuals with similar aberrant phenotype were also detected in flies exposed to TiO_2_ [45]. These results are alarming and underline the necessity of standardized and powerful toxicological characterization protocols for testing nanomaterials in order to select those which respect the safety first principle, with no hazardous effects for human health and environment.

Ag, TiO_2_, Fe_3_O_4_, Au NPs, and carbon nanotubes (single-wall carbon nanotubes—SWCNTs, and multi-walled carbon nanotubes—MWCNTs) were investigated for tissue-specific nanomaterial assessment through the direct microtransfer of nanomaterials into target tissues. This technique assures a gentler and constant release of nanomaterials to the desired location, with no disruption of target tissues and the risk of false-positive results [32]. The microtransfer of different NPs into *D. melanogaster* embryos decreases the viability, allowing to establish a threshold for the minimal toxic dose, as well as the maximal concentrations admitted in the environment. The two highest microtransferred amounts (*i.e.*, 0.10 and 10 ng) of Au NPs, and the three highest microtransferred amounts for TiO_2_ NPs (0.10, 5.1, and 10 ng) and for SWCNTs (7.2 × 10^−4^, 0.07, and 7.2 ng) significantly decreased *D. melanogaster* embryo viability, immediately after microtransfer. SWCNT showed higher toxicity than MWCNT, similar to previous studies [32,56]. The SMART evaluation of the effects of different concentrations of MWCNT showed no DNA damage, as demonstrated by the good survival rates (>90%) and no significant changes in mutation and recombination rates [57]. The most acute toxicity has been induced by TiO_2_ and was most likely caused by the oxidative stress induced by reactive oxygen species produced by TiO_2_ [58].

In the general case of different NPs, toxicity could be the result of both chemical interactions between the biological targets [59] and the nanomaterial and physical obstruction. The increased concentration could favor the NPs aggregation forming large clusters which would diminish the surface to volume ration and therefore affect the environment [32].

Zinc oxide (ZnO) NPs proved to be non-genotoxic in the wing-spot test, but in the comet assay on larvae haemocytes, a significant increase in DNA damage was observed for high doses [60]. Significant changes in Hsp70 and p53 gene expression were also detected [61].

In another study, the mutagenic potential of amorphous ZnO and ZnO NPs was assayed using the wing Somatic Mutation and SMART. Although in the standard cross the two substances were not mutagenic, in the presence of high doses of ZnO NPs a significant increase in the number of mutant spots, generated by mitotic recombination, rather than mutational events, occurred [62].

Copper oxide (CuO) NPs caused significant dose-dependent increases in DNA damage in the comet assay performed on larval haemocytes and also the frequency of mutations in the wing-spot assay, indicating that they are genotoxic, these effects being mediated by oxidative stress [63]. On the other hand, administration of Cu-doped ZnO NPs (3%) in concentrations of 1–8  mg/mL revealed no loss in the climbing and activity behaviours, nor affected the levels of AChE, GSH, GST, LPO, caspase 9/3 and total protein content, suggesting that they are non-toxic [64].

A comparison between Cu and Ag NPs revealed that Cu NPs slow the development, reduce adult longevity and decrease sperm competition in *D. melanogaster*. Ingestion of nanostructured silver decreased larval and pupal survival as well as larval climbing ability, but did not impact on adult longevity and reproductive rate. Interestingly, Ag NPs also reduced the gut microbiota diversity of treated larvae [65]. The changes induced by ingested AgNPs in the fly microbiome have been shown to be due to the differential killing of Ag-susceptible gut bacteria and their replacement by Ag NPs tolerant species. This action could be explained on one side to the wide spectrum intrinsic antimicrobial activity of Ag [66], but also to the induction of a pro-inflammatory effect, interfering with the bacteria-host cross-task. This effect raises questions about the possible indirect implications of AgNPs in the etiology of a multitude of microbiome-associated diseases, such as autoimmune disorders, obesity, type 1 diabetes, autism, multiple sclerosis, and schizophrenia [67]. The partial protection of gut microbial community by anti-oxidant supplementation reveals the role of oxidative stress in the loss of gut microbes and Ag NPs’ toxicity [16].

The comparative genotoxic evaluation of TiO_2_, zirconium oxide (ZrO_2_) and aluminum oxide (Al_2_O_3_) NPs has been performed on third instar larvae fed with NPs and no significant genotoxic activity in the wing spot assay was obtained [68]. 

Cerium oxide (CeO_2_) NPs have been shown to be internalized by the intestinal barrier and haemocytes, but exhibited no toxicity or genotoxicity, although a significant expression of Hsp genes was detected. Moreover, CeO_2_NPs significantly reduced the genotoxic effect of potassium dichromate and the intracellular ROS production [69].

Cobalt (Co) NPs induced significant increases in the frequency of *D. melanogaster* mutants in the wing-spot assay, mainly via the induction of somatic recombination [70].

Synthetic amorphous silica (SiO_2_) with different sizes ranging from 6 to 55 nm fed to larvae in concentrations of 0.1–10 mM induced no significant increases in the frequencies of mutant spots, but an important dose-dependent increase in the levels of primary DNA damage, particularly when high doses (>5 mM) were used [71]. The uptake of SiO_2_ NPs (<30 nm) in the third instar larvae of *D. melanogaster* midgut via endocytic vesicles and by direct membrane penetration (as revealed by TEM) was followed by a significant increase in the expression of hsp70 and hsp22 as well as caspases activation, followed by mitochondrial membrane destabilization and potential loss. Therefore the industrial use of SiO_2_ NPs as a food-additive may represent a human health risk factor [72]. A synthesis of the effects of various NPs categories on different developmental stages of *D. melanogaster* is presented in Table 1.

## 4. Use of *D. melanogaster* Model for the Study of NPs-Based Delivery Systems

The fly model can be used to evaluate the development of nanoparticle-based drug or gene delivery system. In order to be used as a therapeutic delivery system, the NPs should not be cytotoxic to the organism and must be effectively incorporated into the target tissue, without affecting its normal functions. It has been shown that organically modified silica (ORMOSIL) NPs, showing a promising potential as nanoprobes for imaging diagnosis, are biocompatible and have no deleterious effects on the brain tissues or neuronal processes after incorporation into living larval neuronal tissues [73,74]. The larval nervous system of *D. melanogaster* exhibits a high degree of morphological and functional similarity to vertebrates. In order to test the biocompatibility of ORMOSIL in a living organism, different phases of the life cycle of the fly were investigated. At room temperature (25 °C), 1st instar larvae hatched within 24 h after fertilization, then undergo three one-day lasting larval stages, followed by metamorphosis, pupation, and adult flies occurrence after 4–5 days of pupation. As adult flies may live for over 100 days (when maintained at 18 °C) this allows using the fly model for aging studies. The NPs have been delivered by ingestion for about 40 days of the life cycle. The larval stages were assessed for locomotion behaviors and survival rates. The number of pupae that pupated and of adults was counted and the developmental time was also monitored. Adult flies were evaluated for 100 days for flying and synapse transmission defects (bang sensitive test) [75]. The short-term survival of dissected larvae, evaluated by twitching phenotype was not affected by ORMOSIL NPs. *D. melanogaster* model was also used to assess the effects of nano-alumina on the central nervous system using patch clamps. Nano-alumina proved to interact with the rhythmic activities in the antennal lobe of *D. melanogaster* by decreasing the average frequencies of spontaneous activities [76]. 

**Table 1:** The effects of different types of NPs on *D. melanogaster* experimental model.

NPs	Concentrations of NPs	Developmental Stage at the Time of Exposure	Effects	References
C nanotubes	n/a	larval	tissue incorporation, no toxic effects	[33,34,35,56,57]
		adult	affected grooming that resulted in impaired locomotor function and mortality	
GaP nanowires	n/a	larval/adult	no incorporation	[36]
Fe_3_O_4_	n/a	adult	compromised fecundity	[37]
			compromised oogenesis	
			ovarian defects	
			developmental delay	
TiO_2_	0.002–2 mg/L	larval	increased pupation time	[41,42,45,46,58]
			catalase and superoxide dismutase 2 down-regulation	
			rare aberrant eye phenotype: “nanomaterial mutated”	
	80–1600 mg/L	larval/adult	cytotoxic effects on midgut and imaginal disc tissues	
			increased DNA damage	
Ag	n/a	larval	oxidative stress	[38,39,47,51,52]
			Hsp70, p53, p-38, caspase-3, and caspase-9 down-regulation	
			reduced larval and pupal survival	
			affected larval climbing activity	
			pigmentation d-efects in adults	
			affected locomotor ability in adults	
			reduced gut microbiota diversity	
	20 mg/L	larval	over 50% pupal lethality	
	up to 50 mg/L	adult	loss of melanin	
			compromised fertility	
			affected vertical movement	
			tyrosinase and superoxide dismutase decreased activity	
	0.1–1 mg/L	embryo to adult	decreased life-span	
	5 mg/L	embryo to adult	compromised fertility	
Au	0.5–2 nM	larval	no toxic effects	[53,54,55]
	5 nM	larval	increased lipid anabolism	
	2.5 mg/L	embryo to adult	aberrant eye phenotype: “nanomaterial mutated”	
ZnO	n/a (high doses)	larval	increased DNA damage	[60,61,62]
			affected Hsp70 and p53 expression	
			increased mitotic recombination	
CuO	n/a	larval	increased DNA damage	[63,64,65]
			cytotoxic effects	
			slowed development	
			reduced adult longevity	
			decreased sperm competition	
CeO_2_	n/a	larval	no toxic effects	[66,69]
Co	n/a	larval	cytotoxic effects	[70]
			increased mitotic recombination	
Silica	n/a	larval	Hsp70, Hsp22, and caspase up-regulation	[71,72,73,74]
			membrane destabilization	
			mitochondrial membrane potential loss	
	0.1–0.5 mM	larval	reduced toxic effects	
	>5 mM	larval	increased DNA damage	
Alumina	n/a	adult	decreased average frequencies of spontaneous rhythmic activities in the antennal lobe	[76]

It has been shown that the *p*-aminobenzoic acid (PABA) conjugates can serve as drug delivery systems for the oral delivery of NPs in the *D. melanogaster* model [77]. These organic nanocomposites proved to have negligible adverse effect on cellular physiology, behavior, sensitivity to adult sex and other pharmacokinetics parameters of *D. melanogaster*. In live insects, following oral feeding the nanomaterials induced a systemic response due to gut peristaltism. Consequently, the nanomaterials cross the cell membrane barrier and enter into cytoplasm by energy dependent endocytosis. The nanomaterials carrying C_11_ and C_16_ acid side chains are best suited for optimal entry in cells and multiple organs [78].

Self-assembling NPs of amphiphilic polymers can transport hydrophobic molecules across hydrophilic media. The use of a photoactivatable fluorescent cargo (e.g., borondipyrromethene chromophore and a photo-cleavable oxazine) could allow the real-time optical control and tracking of the nanocarrier translocation across different media, including *D. melanogaster* embryos for long periods of time with no cytotoxic effects [79]. For example, poly(2-aminoethyl methacrylate) and poly(2-(dimethylamino)ethyl methacrylate)star polymers have been synthesized with perylene diimide as the central fluorophore in order to obtain a macromolecular carrier with fluorescent tracing and imaging properties during gene delivery. The two polymers delivered DNA into live cells with higher efficiency and low cytotoxicity [80]. *D*. *melanogaster* model proved to be useful for assessing the efficiency of genetic transformation technology using plasmid DNA encapsulated into different nanomaterials to assure a better protection against nucleases. It has been shown that free plasmid DNA with linear polyethylenimine based nanoparticle proved to be more efficient in the *D. melanogaster* based germ-line transformation technology, at a DNA concentration of 0.04 μg/μL [81]. Also, the insect neuropeptide allatostatin 1 from *D. melanogaster* proved to be able to transfect living NIH/3T3 and A431 human epidermoid carcinoma cells and also to transport QDs both in the cytoplasm and nucleus of the respective cells. The conjugation of allatostatin 1 with streptavidin-coated CdSe-ZnS QDs allowed the successful transfection of QD-allatostatin conjugates in the live mammalian cells and the nuclear accumulation of QDs, with the release of allatostatin in an active form, as demonstrated by the increased proliferation rate of transfected cells. The peptide was localized mainly to the cytoplasm, microtubules, and nucleus. These results demonstrate that this conjugate can be used for optimized cell transfection, cellular/nuclear/organelle labeling and for bioactive molecules delivery [82].

## 5. Conclusions

Due to its well-known genetics, developmental and behavioral characteristics, *D. melanogaster* represents an ideal model system for the *in vivo* assessment of NPs’ toxicity, cellular, and subcellular target structures and mechanisms of interaction, providing results that could be reliably extrapolated to mammalian systems and even translated to clinical trials. Besides the assessment of cytotoxicity, *D. melanogaster* shows great promises for investigating the influence of different NPs on the metabolic pathways of mammalian host. With the huge and rapid advancement in the amount of nanomaterials fabricated by different synthesis routes, with variable compositions and morphologies requires a cost-effective, reliable model for *in vivo* assessing of their toxicity. An important experimental perspective should consider the thorough testing of the potential mutagenic activity of various NPs, along with microarray gene expression investigations, in order to evaluate the impact of NPs on *D. melanogaster* genome. The fly model is adaptable for the quick and easy through-put *in vivo* screening assays of nanotoxicity, enabling a proper selection of different nanostructures for a certain biomedical application. 
